# Korean Grammatical Error Correction Based on Transformer with Copying Mechanisms and Grammatical Noise Implantation Methods

**DOI:** 10.3390/s21082658

**Published:** 2021-04-10

**Authors:** Myunghoon Lee, Hyeonho Shin, Dabin Lee, Sung-Pil Choi

**Affiliations:** Department of Library and Information Science, Kyonggi University, Gyeonggi-do 16227, Korea; vhxmtpqms@naver.com (M.L.); shinhh9554@gmail.com (H.S.); leedabin0616@gmail.com (D.L.)

**Keywords:** Grammatical Error Correction (GEC), Neural Machine Translation (NMT), transformer, Copying Mechanism

## Abstract

Grammatical Error Correction (GEC) is the task of detecting and correcting various grammatical errors in texts. Many previous approaches to the GEC have used various mechanisms including rules, statistics, and their combinations. Recently, the performance of the GEC in English has been drastically enhanced due to the vigorous applications of deep neural networks and pretrained language models. Following the promising results of the English GEC tasks, we apply the Transformer with Copying Mechanism into the Korean GEC task by introducing novel and effective noising methods for constructing Korean GEC datasets. Our comparative experiments showed that the proposed system outperforms two commercial grammar check and other NMT-based models.

## 1. Introduction

**Figure 1:**
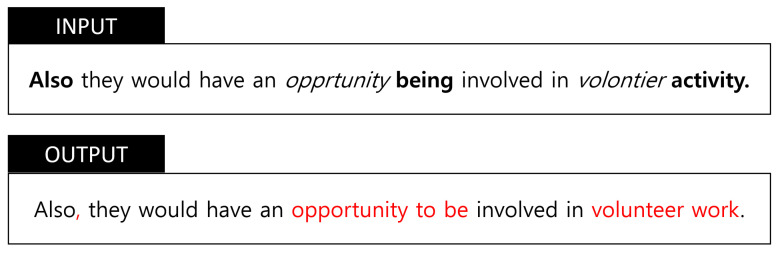
Grammatical Error Correction (GEC) Example.

Grammatical Error Correction (GEC), as shown in Figure 1, is the task of automatically detecting and correcting various types of grammatical errors and typos in texts. It typically focuses on all the textual mistakes and errors including morphological, lexical, syntactic, and semantic irregularities that could be appeared in texts [1].

Until now, almost all the previous approaches to GEC for Korean have utilized the rule-based methods where all the target error patterns as well as corresponding correction logics should be recognized in advance and consistently expanded [2]. However, it is obvious that the rule-based mechanisms have a disadvantage in that they require much of manual labor in achieving the error patterns and correction logics. Furthermore, it is unlikely to promptly reflect a radical change in the current linguistic environment such as the rise of newly coined words and the natural extinction of old-fashioned words and syntactic rules [1].

To address the limitations and problems mentioned earlier, many researchers are now attempting to apply Neural Machine Translation (NMT) models for the GEC because they are perfectly appropriate for the task translating grammatically incorrect sentences to correct sentences. The NMT-based models have two advantages. Firstly, their neural encoder-decoder mechanism effectively encodes various grammatical errors in training data and generates the corresponding corrected texts based on the encoded information [3]. In addition, their error handling coverage is much broader than the conventional methods even handling infrequent and rare error patterns with the generalization ability of the mechanism [1]. These strength of the models leads to the remarkable performance improvements in the recent English GEC tasks showing the promising potentials of the approaches as a future research direction [3].

In this paper, we introduce an effective Korean Grammatical Error Correction model based on Transformer equipped with the Copying Mechanism and various noising methods for automatically generating a training set. Transformer is a model derived from “Attention is all you need,” a paper published by Google in 2017. It follows the existing seq2seq structural encoder-decoder, but it is a model implemented only with Attention as the name of the paper [4]. It is shown that during the GEC execution, about 80% of input texts remain unchanged and only 20% are recognized as errors and thus the system changes their lexical and syntactic structures. The Copying Mechanism can effectively cope with the phenomenon by enhancing the preservation capability of the Transformer [5,6]. Following the promising results of the English GEC task, we apply the Transformer with Copying Mechanism into the Korean GEC task by introducing novel and effective noising methods for building Korean GEC datasets. In the case of the current Korean language, since there is no officially released GEC parallel corpus data, only the data generated by the noising methods were trained and tested for the model. Our contributions are summarized as follows:

- We introduce a novel approach to create Korean GEC datasets by implanting various realistic grammatical errors appearing in Korean texts into original correct sentences and thus capable of creating Korean parallel corpora for GEC in an effective manner.
- We implemented a Transformer-based Korean GEC engine equipped with the Copying Mechanism and a realistic grammatical error detection and correction rule set for many errors that cannot be handled by the main model.
- We showed that the proposed system drastically outperforms two commercial GEC engines in various aspects.
- We analyze the results by comparing the performance with other NMT-based models.

## 2. Related Work

Recently, many studies have been conducted on grammatical error correction models based on neural machine translation [1]. The early stages of the research on the NMT-based GEC mainly focused on LSTM-based encoder/decoder [7]. The introduction of the attention mechanisms into the sequence-to-sequence models [8] improves the performance of the GEC [9].

With Transformer [4] actively exploited in many NLP areas, the recent NMT-based GEC approaches are now adapting the Transformer instead of the traditional RNN-based encoder-decoder models and enjoying their competitive and promising performance compared to the conventional architectures [10,11]. The Copying Mechanism introduced for the machine translation for preserving unknown and special words appeared in source sentences [5] was applied to the GEC models and showed the improved performance in ACL BEA 2019 [12].

The current studies of the NMT-based GEC for Korean language are severely suffering from the lack of the necessary parallel corpora, which makes it very difficult to develop and improve their systems unlike the English GEC. Recently, grammatical noise implantation methods are facilitating the automatic construction of the parallel corpora for the Korean GEC while there is no systematic and effective approach to the noising models specialized for Korean language. Several recent initial attempts are now trying to build the parallel corpora and utilize the Transformer for Korean GEC [3,13]. In the case of China, which is an East Asian cultural region such as Korea, there is a lack of corpus to be used for GEC learning, like Korean. So, in the case of Zhao and Wang [14], the method of giving noise was overcome by applying the dynamic masking technique.

## 3. Methods

**Figure 2:**
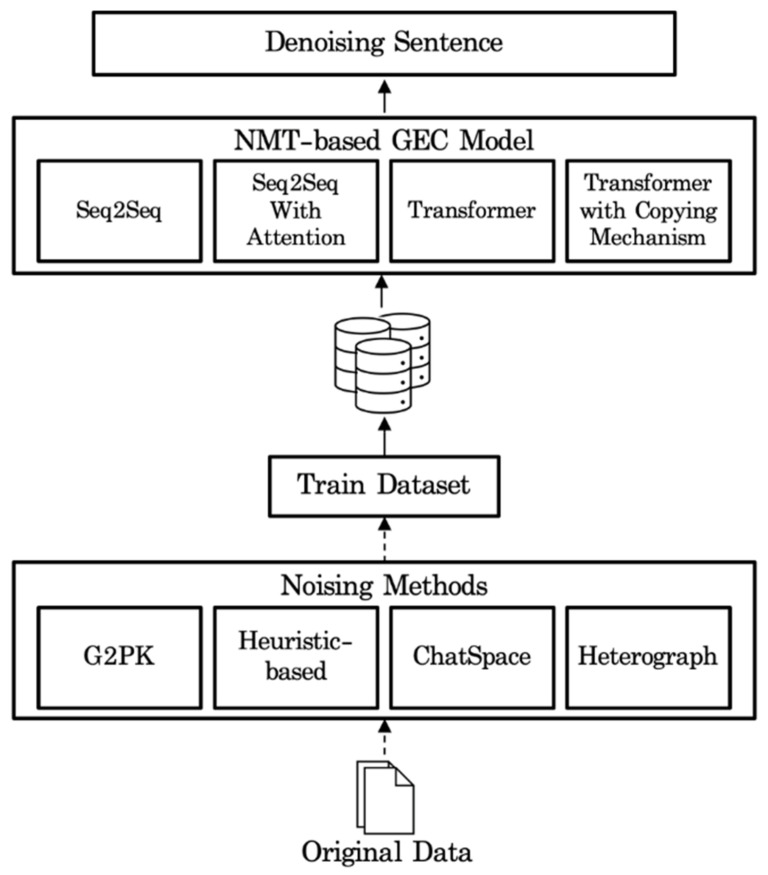
Method Pipeline. NMT, Neural Machine Translation.

We introduce four noising methods for automatically generating a training dataset and a Korean GEC model based on Transformer with Copying Mechanism as shown in Figure 2. 

The training dataset is created by four noise generation methods consisting of Grapheme to phoneme, Heuristic-based, Word spacing, and Heterograph noising rules. Grapheme to Phoneme Noising Rules automatically generate Korean spell errors applying the Korean pronunciation rules. Heuristic-based Noising Rules automatically generate grammatical errors that Koreans are often mistaken. Word spacing noising rules generate spacing errors through the ChatSpace model, and Heterograph noise rules generate grammatical error by converting a word into another form of a word that is similarly pronounced. We train the models of Sequence to Sequence (Seq2Seq) [15], Seq2Seq with Attention [8], Transformer [4], and Transformer with Copying Mechanism model [5] with the data set to compare and analyze the performance. Seq2Seq is an end-to-end model based on a recurrent neural network (RNN) and is composed of an encoder-decoder structure. The encoder transforms the input sequence into a context vector using an RNN-based model, and the decoder converts this context vector into an output sequence. However, the RNN-based Seq2Seq model has a disadvantage in that some information is lost in the process of converting an input sequence into a vector. The Seq2Seq with Attention model tried to solve the problem of the Seq2Seq model by using attention, but it was not completely solved. Unlike the Seq2Seq model, Transformer is a machine translation model that use only self-attention without using RNN-based model and widely used in GEC task. Transformer with Copying Mechanism is a model that improves performance by adding a Copying Mechanism to the Transformer model to enable training for the generation mechanism and the copy mechanism for words in the input sentence, respectively. Our main model is Transformer with Copying Mechanism model, so we focus on that model.

### 3.1. Grammatical Noise Implantation for Korean Language

#### 3.1.1. Grapheme to Phoneme Noising Rules

The complicated pronunciation rules for Korean language lead to the radical and clear difference between its written texts and their pronunciations. The phenomenon causes various lexical errors when writing Korean sentences. One of the pronunciation rules causing errors is “linking sound rule.” The linking sound rule is a phonological phenomenon in which the ending sound of the preceding syllable becomes the first sound of the latter syllable when a syllable that ends with a consonant is followed by a formal morpheme that begins with a vowel [16]. Normally, many people make a mistake by confusing the right words and sentences with their pronunciation, especially produced by the linking sound rule as shown in Table 1. “오랜만에” is a Korean adverb that means “A long time,” but “오랜마네” is a non-existent word and has no meaning.

The noise rules were constructed by using Grapheme to Phoneme module for Korean (G2PK) [17] that can automatically generate Korean spell errors applying the above pronunciation rules. Table 2 shows a Korean sentence generated by the G2PK, in which the correct word, “밥을” is pronounced as “bab-eul” and the incorrect (noised) word, “바블” is sounded as “babeul” artificially generated by the G2PK. Words marked in blue in Table 2 are non-existent words and are the same in all tables.

#### 3.1.2. Heuristic-Based Noising Rules

Korean language is morphologically agglutinative, and a word is composed of its component morphemes. Moreover, a single syllable typically consists of an initial, medial, and final consonant, which complicates the entire language system even more. These complications cause many people using Korean as their mother tongue to make various mistakes in writing texts. Korea has a history of Japanese colonization, so there are some cases where some Koreans use Japanese grammar and words without knowing whether they are grammatical errors. In addition, in Korean, there are many borrowed words written in Korean using the English pronunciation as it is. An example of English is a tsunami from Japanese. Furthermore, like other languages, Korean is also changing continuously in that newly coined words are created, and its grammatical system is also modified reflecting the current linguistic environment.

In this paper, to reflect this situation of Korean, grammar and spelling error rules that Koreans often miss are constructed. Examples of grammatical errors that Koreans easily commit were collected through Internet materials such as Korean language regulations published by the National Institute of the Korean Language, misuse of broadcast and newspaper companies, newspaper articles, and Wikipedia. The collected cases were categorized into about 120 rules and organized. Of the 120 cases, spelling errors were generated by constructing an error dictionary, and grammatical errors were constructed through regular expressions and Python coding to generate errors in the original sentence.

Examples of heuristic errors are “깨끗이” and “깨끗히” as shown in Table 3. “깨끗이” is a Korean adverb that means “Cleanly.” “깨끗히[kkaekkeushi],” marked in blue, is a non-existent word and is an inscription expression like “깨끗이[kkaekkeus-i]” pronunciation. Some Koreans are often mistaken in the writing process because the pronunciation of these two words is similar.

Other examples of heuristic errors are “오랜만에[olaenman-e]” and “오랫만에[olaesman-e].” “오랜만에” is a Korean adverb that means “A long time.” “오랫만에” is a grammatically incorrect expression. However, some Koreans often use the word “오랫동안 [olaesdong-an]” (For a long time) in a similar manner.

#### 3.1.3. Word Spacing Noising Rules

In Korean, the rules of spacing are complicated, so college students who have a higher education are often wrong [18]. In order to deal with word spacing errors, we also generate word spacing noises by using ChatSpace [19]. ChatSpace is an automatic Korean word spacing package, although its performance is not so good in practice, as underlined in Table 4.

We exploit the imperfect behavior of the ChatSpace. First of all, an input sentence is passed through the ChatSpace model with all spaces removed. ChatSpace should perform the word spacing with the input and make some mistakes in the process. We consider these mistakes as the word spacing noises.

#### 3.1.4. Heterograph Nosing Rules

Heterograph refers to words that have the same or similar pronunciation but have different spellings. In this paper, it is limited to syllable units, not words, and in English, the pronunciation is the same as “peə(r),” but the different spellings are “Pair” and “Pear” as heterographs.

In order to generate a heterograph error, the syllables with the same phonetic symbol or similar phonetic symbol as shown in Table 5, were classified as neutral and final with reference to Roman pronunciation notation. In the case of neutral, the syllables with [a] added to the phonetic symbol were judged to have a similar phonetic symbol, and in the case of the final, the syllables with the same phonetic symbol or repeated phonetic symbols were judged to have similar phonetic symbols.

As can be seen in Table 6, a grammatical errors is generated by replacing “ㄱ[k]” and “ㅆ[tt]” at each final position in “먹-[meok-]” and “-었-[-eott-]” with “ㄲ[kk]” and “ㅅ[t].” “먺엇[meokk-eot]” is non-existent word.

### 3.2. Transformer

Our system is based on the attention-based Transformer architecture in which has an encoder and decoder as atomic modules. Each encoder and decoder consist of a multi-head self-attention layer followed by a position-wise feed-forward layer, along with residual connection and layer normalization [4]. Unlike the encoder, decoder consists of a total of three sub-layers, two of which are the same as the encoder’s sub-layer, and the other is a sub-layer that calculates multi-head attention for the output of the encoder. Transformer input embedding is combined with a positional embedding and the token embedding in the input sequence.

### 3.3. Copying Mechanism

Copying Mechanism has proven to be effective for text summarization and semantic parsing [5]. Copying Mechanism is added to the end of the Transformers. The output probability distribution of the Copying Mechanism is a mixture of $p^{gen}\;$ and $p^{copy}$. $p^{gen}\;$ is distribution generated from the decoder. $p^{copy}$ is copy distribution, which is defined as the layer of copy attention that assigns a distribution for tokens that appear in the input sentence $\alpha_{t}^{copy}$, which plays the most important role in the Copying Mechanism, defined per each decoding step. $\alpha_{t}^{copy}$ is a balance factor that decides whether to reflect the distribution of the input sentence or the distribution generated by the Transformer. It is calculated through the copy scores $A_{t}^{T}$, which is the output of the copy attention, and the value V of the copy attentions hidden state.

(1) $$\alpha_{t}^{copy}\;=\;sigmoid\left(W^{T}\sum \left(A_{t}^{T}\cdot V\right)\right)$$

(2) $$P\left(y_{t}\right)=\left(\;1-\;\alpha_{t}^{copy}\right)*\;P^{gen}\left(y_{t}\right)+\;\alpha_{t}^{copy}*\;P^{copy}\left(y_{t}\right)$$

**Figure 3:**
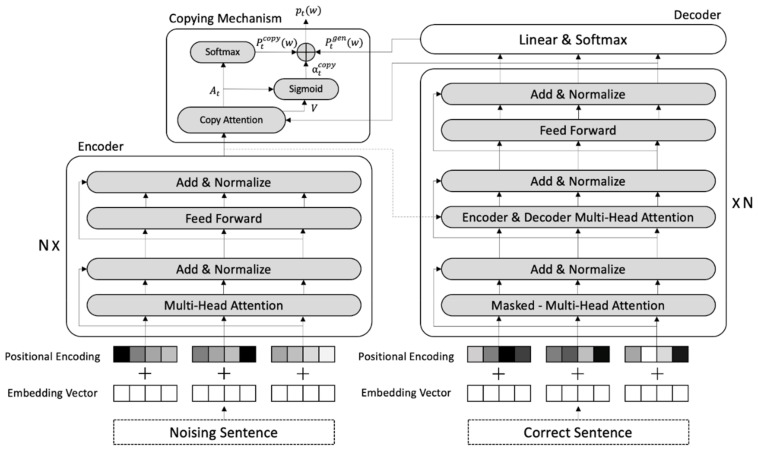
Model Architecture.

As shown in the formula above, if the $\alpha_{t}^{copy}$ value is greater than 0.5 it reflects copy distribution more in the final distribution value, and if it is less than 0.5, it reflects generation distribution. The finally computed distribution determines the word with a high probability as the word in the output sentence [5]. The final architecture of our GEC model is shown in Figure 3.

## 4. Experiments and Discussion

In this paper, a Korean GEC experiment was conducted by comparing the performance of two commercial GEC engines and NMT-based GEC models. Commercial GEC engines are Py-Hanspell (Naver API) [20] and Hanspell (Kakao API) [21] provided by portal sites most used in Korea, respectively, and are currently available for free as a beta service. The performance was measured through Precision, Recall, F0.5-score, BLEU [22], and GLEU [23].

### 4.1. Data

By applying the previously mentioned noising rules, we constructed a parallel dataset for the Korean GEC by using AI-Hub Korean-English parallel corpus [24] released by NIA. The dataset includes 1.1 million Korean-English literary-style sentence pairs and 500K colloquial sentence pairs. Table 7 shows the detailed information of the dataset.

The dataset includes 1,600,000 sentences from various domains such as news articles, web pages, formal documents, and even daily conversations, which reflects broad linguistic aspects. We applied the grammatical noise implantation rules into the dataset and generated a large set of sentence pairs for the Korean GEC. For the experiments, we generated 6,409,672 sentence pairs of noise implanted sentences and original ones. Each noise method was applied to the original sentence. In addition, sentences that do not generate errors because there is no noise rule in the original sentence were also configured in the data set. The reason is that not everyone uses only the wrong sentences in the spell checker, and when the model receives the correct sentence, it has to be returned as it is. A total of 4,486,756 pairs were used for the training set and 640,956 and 1,281,960 pairs were used for the development set and test set, respectively.

### 4.2. Model and Parameters

Our GEC model uses a typical configuration of the Transformer with Copying Mechanism in that all the input tokens are embedded and encoded by the conventional positional encoding mechanism. As shown in Table 8, we use a 4096-dimensional position-wise feed-forward layer. In addition, both the token embedding size and hidden size are 512. For the Copying Mechanism, we apply a single layer with eight attention heads. Adam optimizer was used in the training. The batch size during training was set to 100 and the dropout ratio and label smoothing value were all set to 0.1. We trained our own tokenizer by using SentencePiece [25] where the size of the source (encoder) and target (decoder) vocabulary was set to 30,000. In this study, in order to prevent overfitting of the model, early stopping was performed when there was no improvement in the performance of the verification data for three epochs during the training process.

### 4.3. Evaluation Metrics

To evaluate the model’s performance, GLEU (Generalized Language Evaluation) [23], BLEU (Bilingual Evaluation Understudy) [22] and F0.5 scores were used. BLEU, which is often used to evaluate machine translation models, derives performance by calculating the similarity between system prediction results and reference data. In this paper, BLEU1 ~ BLEU4 were calculated and evaluated. BLEU can be used regardless of language and has a fast calculation speed, and higher means better performance. The GLEU metric is a variant of BLEU proposed for evaluating grammatical error corrections using n-gram overlap with a set of reference sentences, as opposed to precision/recall of specific annotated errors [23]. Like BLEU, GLEU shows better performance with higher numbers.

F0.5 is a performance evaluation that emphasizes precision rather than recall. On the GEC, task recall is calculated as the percentage of correct predictions for the positive class out of all positive predictions, indicating the proportion of the actual corrected sentences among the total grammatical error sentences. Precision refers to the proportion of sentences with grammatical errors among the corrected sentences by calculating the percentage of correct predictions for the positive class. In the case of GEC task, finding the wrong part and correcting the wrong part are both important, but using F0.5 means, more importantly, whether the wrong part is corrected properly.

### 4.4. Result and Discussion

Table 9 shows the comparative results of the proposed system and the other models by using both BLEU and GLEU scores. The bold text in the Table 9 indicates the best performance in the experiment.

As can be seen from Table 9, the model presented in this study outperforms other NMT-based models and two commercial grammatical error correctors. In particular, NMT-based models are ahead of the performance of the two commercial grammar services with GLEU and BLEU scores. In addition, in every part of score, our model outperforms than Seq2Seq, Seq2Seq with Attention, and Transformer models.

Table 10 shows the detailed evaluation results of the systems denoting precision, recall and F0.5 scores by using our test data mentioned earlier, and the bold text is the best performance model in the experiment. Our grammatical noise implantation method mainly reflects typically and frequently committed grammatical errors that all the conventional grammar checking, and correcting systems should handle effectively. Therefore, the comparison using the test set seems to be fair and objective. Table 10 shows similar results to the BLEU and GLEU performance evaluation. The model presented in this study outperforms other NMT-based models and two commercial grammar error correctors and shows a large difference in performance when compared to a commercial grammar service. In addition, the model using the Copying Mechanism shows higher performance in Precision, Recall, and F0.5 than that of Transformer.

Through the results, it was confirmed that the Korean grammar correction performance of the Transformer with Copying Mechanism model applied was the highest. The Seq2Seq model using the existing Bi-LSTM showed lower performance than the models capable of parallel processing. This is because the Seq2Seq model using Bi-LSTM tends to forget the data at the beginning of the input, and the performance decreases as the length of the sentence increases. In the case of the model to which the attention was applied, the above problem was partially solved, but when looking at the results of this experiment, the above model was still not completely overcome. Unlike the Seq2Seq model, the Transformer solved the above disadvantages by using self-attention rather than using the RNN series model, and it can be seen that the results of the experiment show high performance in correcting Korean grammar errors. However, since Transformer approaches the problem from the point of view of generating the entire sentence, it has the disadvantage of copying the word as it is. In the case of the Transformer model with Copying Mechanism applied, the performance was higher than that of the Transformer model because the generating part and the copying part can be trained separately.

Table 11 denotes the outputs of the three systems used in the experiment with an input sentence with various grammatical errors including a pronunciation-related error, contextual error, and word spacing error. In the sentence, the pronunciation-related error is denoted in italic, the contextual error is indicated by boldface, the word spacing error is marked by underscore, and non-existent words are marked in blue. While Py-Hanspell (Naver API) could detect and correct the second word spacing error, it fails to handle all the others. In particular, Py-Hanspell (Naver API) incorrectly revised the first word spacing error suggesting an overly spaced token. Besides this, Hanspell (Kakao API) fails to handle all the errors in the sentence. On the contrary, our system successfully detects, and correct all the errors in the sentence. In particular, our system could detect and correct the contextual error by revising the word “가리켰다 (pointed to)” which is lexically correct but inappropriate semantically to “가르쳤다 (taught).”

Table 12 shows that the Transformer with Copying Mechanism model corrects the grammatical errors constructed in this paper. The words highlighted in Table 12 are the same as in Table 11. 

The first example is the result of correcting grammatical errors generated by the G2PK noise method. The G2PK noise is the phonological phenomenon grammatical error. In the case of example, grammatical error sentences are created by changing “들이 [deul-i]” to “드리 [deuli].” When “들이” is pronounced in Korean, it is expressed as “드리” due to the phonological phenomenon. Our model corrected grammatical errors generated by the G2PK noise method and corrected spacing errors. 

The second example is the result of correcting grammatical errors generated by the heuristic-based noise method. The heuristic-based noise method is created based on rules by investigating grammatical errors that Koreans are wrong. In the case of the example, grammatical errors were generated by changing “깨끗이” and “완전히” to “깨끗히” and “완전이.” “깨끗이 [kkaekkeus-i]” is an adverb meaning “clearly,” and “완전히 [wanjeonhi]” is an adverb meaning “completely.” Some Koreans write these two words as “깨끗히 [kkaekkeushi]” and “완전이 [wanjeon-i].” These two words are not in the dictionary. Our model corrected two heuristic-based grammatical errors that appeared in one sentence, and the spacing error was also fixed. 

The third example is an example of correcting grammatical errors created by the Heterographs-based noise method. The Heterographs-based noise methods provide errors by converting a word into another form of a word that is similarly pronounced. For example, a grammatical error was created by changing “예상하다 [yesanghada]” and “못했다 [mothaetda]” to “얘상하다 [yaesanghada]” and “뫃햏다 [mothaetda].” “예상하다” is a verb meaning “predict,” and “못했다” is a past auxiliary verb of “couldn’t.” Our model corrected the two words in the correct format, and, like other examples, the spacing correction was also corrected at the same time.

The machine translation task creates a sentence in a different language than the input sentence. In contrast, grammatical error correction corrects only some words with grammatical errors, and most of the other words are output the same as the input. Therefore, applying a machine translation model to a grammar correction task can replace words without errors with new ones. Because of this problem, applying a Copying Mechanism that can copy words without errors is more suitable for grammatical error correction. This can be seen in Table 13. In the input sentence, the grammatically correct input word “경기도 [Gyeonggi-do]” (One of the provinces in Korea and the provinces surrounding Seoul) was not generated in the Transformer model. However, Transformer with Copying Mechanism model creates the same as the input statement. In other words, it can be seen that the Transformer model applying the Copying Mechanism is more suitable for grammatical error correction.

## 5. Conclusions

This paper introduced a Korean GEC model based on Transformers equipped with the Copying Mechanism as well as a systematic process for automatically constructing parallel corpus for the proposed model. The process involves four grammatical noise implantation rules reflecting general linguistic mistakes made in writing Korean texts. We conducted comparative analysis experiments with three machine translation models and two commercial grammar correction services. The experimental results indicated that the proposed system outperforms the existing commercial grammar correction services in many perspectives including GLEU, BLEU, Precision, Recall, and F0.5. Our proposed model showed better performance than other machine translation models. In particular, it was confirmed that it has an advantage over Transformer in all performance evaluation methodologies. This means that the Copying Mechanism compensates for the problems encountered in machine translation.

Although we attempted to apply typical and frequent errors and typos in generating our dataset, we still seem to be light on the noising rules covering other grammatical mistakes and semantic misuses in Korean language. Therefore, our future research direction would be the enlargement of the rule set by more intensively inspecting error patterns. By applying the extended rule set, it is necessary to construct more expressive datasets covering almost all the lexical, syntactic, and semantic errors appeared in Korean texts.

## Figures and Tables

**Table 1:** Examples of Grammatical Noise Implantation Rules using the Pronunciation Mechanisms of Korean.

Type	Word & Means	
Original Sentence	Korean	오랜만에
	Pronunciation	olaenman-e
	Meaning	After a long time
Noised Sentence	Korean	오랜마네
	Pronunciation	olaen-mane
	Meaning	-

**Table 2:** Example of Noised Sentence by G2PK.

Type	Sentence and Meaning	
Original Sentence	Korean	나는 어제 밥을 먹었다.
	Pronunciation	naneun eoje bab-eul meog-eottda.
	Meaning	I ate meal yesterday.
Noised Sentence	Korean	나는 어제 바블 먹었다.
	Pronunciation	naneun eoje babeul meog-eottda.
	Meaning	I ate babeul yesterday.

**Table 3:** Example of Heuristic-based Noising Rules.

Type	Sentence and Meaning	
Original Sentence	Koreans	나는 집을 깨끗이 청소했다.
	Pronunciation	naneun jib-eul kkaekkeus-i cheongsohaessda.
	Meaning	I cleaned my house **cleanly**.
Noised Sentence	Koreans	나는 집을 깨끗히 청소했다.
	Pronunciation	naneun jib-eul kkaekkeushi cheongsohaessda.
	Meaning	I cleaned my house kkaekkeushi.
Original Sentence	Koreans	나는 오랜만에 친구를 만났다.
	Pronunciation	naneun olaenman-e chinguleul mannattda.
	Meaning	I met a friend after a long time.
Noised Sentence	Koreans	나는 오랫만에 친구를 만났다.
	Pronunciation	naneun olaesman-e chinguleul mannattda.
	Meaning	I met a friend for a long time.

**Table 4:** Example of Output Sentence of ChatSpace according to Input Sentence.

Type	Sentence and Meaning	
Input Sentence	Korean	나는 그럴 수 없지.
	Pronunciation	naneun geuleol su eobsji.
	Meaning	I cannot do that.
Output Sentence	Korean	나는 그럴수 없지.
	Pronunciation	Naneun geuleolsu eobsji.
	Meaning	I cannot dothat.

**Table 5:** Similar Syllables Group Table.

Syllable Position	Similar Syllables Group
Neutral	ㅔ[e], ㅐ[ae]
	ㅖ[ye], ㅒ[yae]
	ㅚ[we], ㅞ[we], ㅙ[wae]
Final	ㅂ[p], ㅍ[p]
	ㅅ[t], ㅆ[tt], ㄷ[t], ㅌ[t], ㅈ[t], ㅊ[t], ㅎ[t]
	ㄱ[k], ㄲ[kk], ㅋ[k]

**Table 6:** Example of Heterograph Noising Rules.

Type	Sentence and Meaning	
Original Sentence	Korean	나는 간장에 밥을 비벼 먹었다.
	Pronunciation	naneun ganjang-e bab-eul bibyeo meok-eottda.
	Meaning	I ate rice on soy sauce.
Noised Sentence	Korean	나는 간장에 밥을 비벼 먺엇다.
	Pronunciation	naneun ganjang-e bab-eul bibyeo meokk-eotda.
	Meaning	I meokkeot rice on soy sauce.

**Table 7:** Statistics and Elements of the Dataset Used.

Domain	Explanation	Size
News	News text	800 K
Government	Government and Local Government Websites, Publications	100 K
Law	Administrative rules, autonomous laws	100 K
Korean Culture	Korean history and culture contents	100 K
Colloquial	Natural colloquial sentences	400 K
Dialogue	Context/scenario-based conversation set	100 K

**Table 8:** Parameters Size.

Parameters	Size
Position-wise Feed forward layer	4096
Encoder/Decoder Layer size	8
Embedding Size	512
Attention-Head	8
Dropout ratio	0.1
Smoothing value	0.1
Vocabulary size	30,000

**Table 9:** Comparison of GEC Models (GLEU, BLEU).

Model	GLEU	BLEU1	BLEU2	BLEU3	BLEU4
Py-Hanspell	46.55	63.27	48.63	38.18	30.39
Hanspell	48.28	63.95	50.23	40.19	32.36
Seq2Seq	72.18	83.38	74.57	66.82	59.89
Seq2Seq with Attention	77.02	86.34	79.09	72.65	66.72
Transformer	76.09	86.10	77.63	70.47	63.67
**Transformer with** **Copying Mechanism**	**79.37**	**88.00**	**80.78**	**74.67**	**68.58**

**Table 10:** Comparison of GEC Models (Precision, Recall, F0.5).

Model	Precision	Recall	F0.5
Py-Hanspell	28.73	28.03	27.81
Hanspell	30.85	29.82	29.80
Seq2Seq	65.88	65.56	65.61
Seq2Seq with Attention	70.94	70.46	70.76
Transformer	73.83	72.46	73.24
**Transformer with** **Copying Mechanism**	**75.30**	**74.13**	**74.86**

**Table 11:** Error Correction Results by Four Systems.

Type	Sentence and Meaning	
Input Sentence with Grammatical Errors	Korean	수업*시**가네* 선생님이수학을 **가리켰다**.
	Pronounciation	sueobsigane seonsaengnim-isuhak-eul galikyeossda.
	Meaning	sueobsigane, my teachermath**pointed to**.
Py-Hanspell	Korean	수업 시 가네 선생님이 수학을 **가리켰다**.
	Pronounciation	sueob si gane seonsaengnim-i suhak-eul galikyeossda.
	Meaning	sueob si gane, my teacher **pointed to** math.
Hanspell	Korean	수업시가 네 선생님 이수학을 **가리켰다**.
	Pronounciation	sueobsiga ne seonsaengnim isuhak-eul galikyeossda.
	Meaning	sueobsiga, your teacher **pointed to**isuhak.
NMT-based models	Korean	수업 시간에 선생님이 수학을 가르쳤다.
	Pronounciation	sueob sigan-e seonsaengnim-i suhak-eul galeuchyeossda.
	Meaning	In class, my teacher taught math.

**Table 12:** Example of Output of Transformer with Copying Mechanism for each Noising Method.

Noise			Sentence and Meaning
G2PK	Input	Korean	그러면 중국사람 *드리*투표를 해줘야 하는데 다소 어렵다.
		Pronunciation	geuleomyeon jung-gugsalam deulitupyoleul haejwoya haneunde daso eolyeobda.
		Meaning	Then, the Chinese have to deulitupy, which is a bit difficult.
	Predict	Korean	그러면 중국 사람들이 투표를 해줘야 하는데 다소 어렵다.
		Pronunciation	geuleomyeon jung-gug salamdeul-i tupyoleul haejwoya haneunde daso eolyeobda.
		Meaning	Then, the Chinese people have to vote, which is a bit difficult.
	Correct	Korean	그러면 중국 사람들이 투표를 해줘야 하는데 다소 어렵다.
		Pronunciation	geuleomyeon jung-gug salamdeul-i tupyoleul haejwoya haneunde daso eolyeobda.
		Meaning	Then, the Chinese people have to vote, which is a bit difficult.
Heuristic	Input	Korean	항상 요리하기 전에 찬물로퀴 노아를 *깨끗히* 씻고 *완전이* 말려라.
		Pronunciation	hangsang yolihagi jeon-e chanmullokwi noaleul kkaekkeushi ssisgo wanjeon-i mallyeola.
		Meaning	Always wash Noah thoroughly with chanmullokwi and dry thoroughly before cooking.
	Predict	Korean	항상 요리하기 전에 찬 물로 퀴노아를 깨끗이 씻고 완전히 말려라.
		Pronunciation	hangsang yolihagi jeon-e chan mullo kwinoaleul kkaekkeus-i ssisgo wanjeonhi mallyeola.
		Meaning	Always wash quinoa thoroughly with cold water and dry thoroughly before cooking.
	Correct	Korean	항상 요리하기 전에 찬 물로 퀴노아를 깨끗이 씻고 완전히 말려라.
		Pronunciation	hangsang yolihagi jeon-e chan mullo kwinoaleul kkaekkeus-i ssisgo wanjeonhi mallyeola.
		Meaning	Always wash quinoa thoroughly with cold water and dry thoroughly before cooking.
Heterograph	Input	Korean	저는 이런 일이 일어나리라고 *얘상*하지 뫃햏거든요.
		Pronunciation	jeoneun ileon il-i il-eonalilago yae sanghaji mothaetgeodeun-yo.
		Meaning	I mothaet yae sang this to happen.
	Predict	Korean	저는 이런 일이 일어나리라고 예상하지 못했거든요.
		Pronunciation	jeoneun ileon il-i il-eonalilago yesanghaji mothaetgeodeun-yo.
		Meaning	I didn’t expect this to happen.
	Correct	Korean	저는 이런 일이 일어나리라고 예상하지 못했거든요.
		Pronunciation	jeoneun ileon il-i il-eonalilago yesanghaji mothaetgeodeun-yo.
		Meaning	I didn’t expect this to happen.

**Table 13:** Example of Output Comparison of Transformer and Transformer with Copying Mechanism for the Correct Sentence.

Type	Sentence and Meaning	
Correct Sentence	Korean	경기도를 나무와 숲으로 둘러싸인 녹색도시로 만들기 위한 특별한 신용카드가 출시된다.
	Pronunciation	gyeonggi-doleul namuwa sup-eulo dulleossain nogsaegdosilo mandeulgi wihan teugbyeolhan sin-yongkadeuga chulsidoenda.
	Meaning	A special credit card is released to make Gyeonggido a green city surrounded by trees and forests.
Transformer	Korean	나무와 숲으로 둘러싸인 녹색도시로 만들기 위한 특별한 신용카드가 출시된다.
	Pronunciation	namuwa sup-eulo dulleossain nogsaegdosilo mandeulgi wihan teugbyeolhan sin-yongkadeuga chulsidoenda.
	Meaning	A special credit card is released to make a green city surrounded by trees and forests.
Transformer with Copying Mechanism	Korean	경기도를 나무와 숲으로 둘러싸인 녹색도시로 만들기 위한 특별한 신용카드가 출시된다.
	Pronunciation	gyeonggi-doleul namuwa sup-eulo dulleossain nogsaegdosilo mandeulgi wihan teugbyeolhan sin-yongkadeuga chulsidoenda.
	Meaning	A special credit card is released to make Gyeonggido a green city surrounded by trees and forests.

## Data Availability

Not applicable.
